# Use of Brain MRI Atlases to Determine Boundaries of Age-Related Pathology: The Importance of Statistical Method

**DOI:** 10.1371/journal.pone.0127939

**Published:** 2015-05-29

**Authors:** David Alexander Dickie, Dominic E. Job, David Rodriguez Gonzalez, Susan D. Shenkin, Joanna M. Wardlaw

**Affiliations:** 1 Neuroimaging Sciences, Centre for Clinical Brain Sciences (CCBS), The University of Edinburgh Medical School, Edinburgh, United Kingdom; 2 Geriatric Medicine Unit, The University of Edinburgh, Royal Infirmary of Edinburgh, Edinburgh, United Kingdom; 3 Scottish Imaging Network, A Platform for Scientific Excellence (SINAPSE) collaboration, Glasgow, United Kingdom; Centre Hospitalier Universitaire Vaudois Lausanne - CHUV, UNIL, SWITZERLAND

## Abstract

**Introduction:**

Neurodegenerative disease diagnoses may be supported by the comparison of an individual patient’s brain magnetic resonance image (MRI) with a voxel-based atlas of normal brain MRI. Most current brain MRI atlases are of young to middle-aged adults and parametric, e.g., mean ±standard deviation (SD); these atlases require data to be Gaussian. Brain MRI data, e.g., grey matter (GM) proportion images, from normal older subjects are apparently not Gaussian. We created a nonparametric and a parametric atlas of the normal limits of GM proportions in older subjects and compared their classifications of GM proportions in Alzheimer’s disease (AD) patients.

**Methods:**

Using publicly available brain MRI from 138 normal subjects and 138 subjects diagnosed with AD (all 55–90 years), we created: a mean ±SD atlas to estimate parametrically the percentile ranks and limits of normal ageing GM; and, separately, a nonparametric, rank order-based GM atlas from the same normal ageing subjects. GM images from AD patients were then classified with respect to each atlas to determine the effect statistical distributions had on classifications of proportions of GM in AD patients.

**Results:**

The parametric atlas often defined the lower normal limit of the proportion of GM to be negative (which does not make sense physiologically as the lowest possible proportion is zero). Because of this, for approximately half of the AD subjects, 25–45% of voxels were classified as normal when compared to the parametric atlas; but were classified as abnormal when compared to the nonparametric atlas. These voxels were mainly concentrated in the frontal and occipital lobes.

**Discussion:**

To our knowledge, we have presented the first nonparametric brain MRI atlas. In conditions where there is increasing variability in brain structure, such as in old age, nonparametric brain MRI atlases may represent the limits of normal brain structure more accurately than parametric approaches. Therefore, we conclude that the statistical method used for construction of brain MRI atlases should be selected taking into account the population and aim under study. Parametric methods are generally robust for defining central tendencies, e.g., means, of brain structure. Nonparametric methods are advisable when studying the limits of brain structure in ageing and neurodegenerative disease.

## Introduction

The structure of the human brain changes with age. Loss of normal grey and white matter (GM, WM) and the appearance of WM hyperintensities are common on magnetic resonance imaging (MRI) in cognitively normal older people. However, these changes in brain structure vary between individuals and their patterns and consequences are not fully understood [1–7]. This variability in brain structure is present even between people of the same age and cognitive function, and the range of variability increases with advancing age [2,8,9]. It is therefore difficult to establish whether brain MRI in any one individual subject is normal for age, or suggestive of neurodegenerative disease, e.g. Alzheimer’s disease [2,3,10,11].

Alzheimer’s disease (AD) is now a major public health burden in many countries as their populations age [12,13]. AD is generally characterised by accelerated loss of GM in the medial temporal lobe, particularly in the hippocampus [14–17]. The earliest stages of pathological GM loss are subtle and difficult to differentiate from normal variation [2,15]. This presents a problem because future treatments are likely to be most effective if administered in the earliest stages of disease [13]. Sensitive and specific methods would help to detect whether subtle changes in brain structure are normal for age or indicative of incipient neurodegenerative disease.

Voxel-based atlases of brain structure, although traditionally used for preprocessing in brain MRI analyses (e.g., registration and segmentation; [18]), would also be useful for highlighting abnormal brain structure in individuals [19–21]. An atlas of the range of normal brain structure in ageing could be used in a similar manner to population derived charts used for monitoring the weights and growth of children [22,23].

Existing voxel-based brain MRI atlases of the structure of the normal brain are largely derived from parametric, e.g., mean ±standard deviation (SD), methods and assumptions [21,24]. Parametric atlases are created by aligning a number of subjects into a standard brain MRI space, e.g. Montreal Neurological Institute (MNI) space; the mean and SD are then calculated in each voxel to estimate the normal range of values for each voxel [24]. For example, the mean ±2 SD is used to estimate the 95% limits of each voxel value. However, the distributions of values within voxels need to be approximately Gaussian (i.e., “statistically Normal”) for these estimates to be robust.

It is unclear whether the distributions of brain MRI voxel values are approximately Gaussian during normal ageing [9,25,26]. Previous work has assessed the impact of non-Gaussian data in group studies of younger subjects and diffusion tensor imaging [27–33]. We found no previous assessment of the impact of non-Gaussian data in individual, atlas-based studies of brain volume or GM in older subjects (≥60 years). Therefore, it remains important to determine if normal GM limits in older subjects are Gaussian or not because excessive GM loss is strongly associated with cognitive decline and AD [15–17].

In this work, we examined publicly available structural brain MRI in normal older subjects (≥60 years) to: firstly, assess the data distributions within GM in old age; secondly, construct a parametric atlas of GM in normal subjects at older ages; thirdly, construct a nonparametric atlas of GM in the same normal older subjects; and finally, compare the abilities of parametric versus nonparametric atlases to classify GM values as normal or abnormal in patients diagnosed with AD.

## Methods

### 2.1 Subjects

Structural brain MRI data were obtained for 49 normal and 49 AD subjects (60–90 years and demographically matched) from the Open Access Series of Imaging Studies (OASIS; http://www.oasis-brains.org/). A further 49 normal subjects and 89 subjects diagnosed with AD (55–90 years and demographically matched) were acquired from the Alzheimer’s Disease Neuroimaging Initiative (ADNI; adni.loni.usc.edu).

Using computerised random number generation, we randomly selected 49 normal older subjects (>60 years) from each database to ensure the normal atlases we created were not biased to either cohort. In total, there were 98 normal older subjects available in OASIS with useable T1-weighted images and 138 from ADNI at the time of this study. We are preparing a separate report which assesses the remaining 49 normal older subjects from OASIS and 89 from ADNI. These additional normal older subjects were not included here because the focus of the current work is assessment of AD subjects using a normal older control atlas that is not biased towards any particular cohort.

We used 49 AD subjects from OASIS and 89 AD subjects from ADNI to determine whether sample size influenced classification results, i.e., whether misclassifications were partly due to chance in smaller test groups. As with normal subjects, AD subjects were randomly selected from each database using computerised random number generation, i.e., a spreadsheet of all subjects was downloaded from the databases and a randomly generated number list was used to randomly select subjects from each group.

The ADNI, through collaboration among government, private, and non-profit organizations (listed in the acknowledgements), recruited subjects from over 50 sites across the United States and Canada to test whether imaging and other biological markers and clinical and neuropsychological assessment can be combined to measure the progression and better treat mild cognitive impairment (MCI) and early AD. For up-to-date information, see www.adni-info.org.

At the time of the present study, ADNI and OASIS were the only public sources of structural MRI brain scans with clinical metadata that fully represented normal older people (≥60 years) [34]. Normal subjects did not have dementia, but potentially had non-debilitating conditions common in ageing, e.g., hypertension. Detailed demographic descriptions were provided previously [9,34].

All subjects used in this work gave written informed consent and the use of these subjects was approved by the Institutional Review Boards (IRB) of ADNI (adni.loni.usc.edu) and Washington University (http://www.oasis-brains.org/).

### 2.2 Brain MRI acquisition and initial processing

In both ADNI and OASIS, 1.5 tesla (T) magnetization prepared rapid gradient-echo (MP-RAGE) T1-weighted MR brain images were acquired in the sagittal plane at approximately 1x1x1mm resolution. The full acquisition parameters are described elsewhere [35,36]. Although 3 T images were available from ADNI, we used 1.5 T images from ADNI because only 1.5 T images were available from OASIS. Different field strengths may have created a bias between the samples.

Non-brain structure was carefully removed from the images by the following steps:

1. Each subject was oriented to the position and angle of the MNI 152 template brain mask (http://www.bic.mni.mcgill.ca/ServicesAtlases/HomePage), using Functional MRI of the Brain’s (FMRIB’s) Linear Registration Tool (FLIRT) [37].
2. A brain mask of each subject was created by diffeomorphically warping the template to each subject [38].
3. The resulting brain mask of each subject was applied to their FLIRT-registered image to remove non-brain structure.
4. The results of steps 1 and 2 were visually inspected by slice and errors, e.g. remaining skull, manually corrected using the Multi-image Analysis GUI (http://ric.uthscsa.edu/mango/download.html).

Next, we performed bias field correction and calculated GM proportion images (voxel-wise GM proportion) using T1 intensity and spatial neighbourhood information [39]. Hypointense areas in WM (hyperintensities on T2) that were incorrectly classified as GM were removed from the GM proportion images via transformation to standard space with manually verified classifications of GM.

### 2.3 Registration target (standard space)

**Fig 1:**
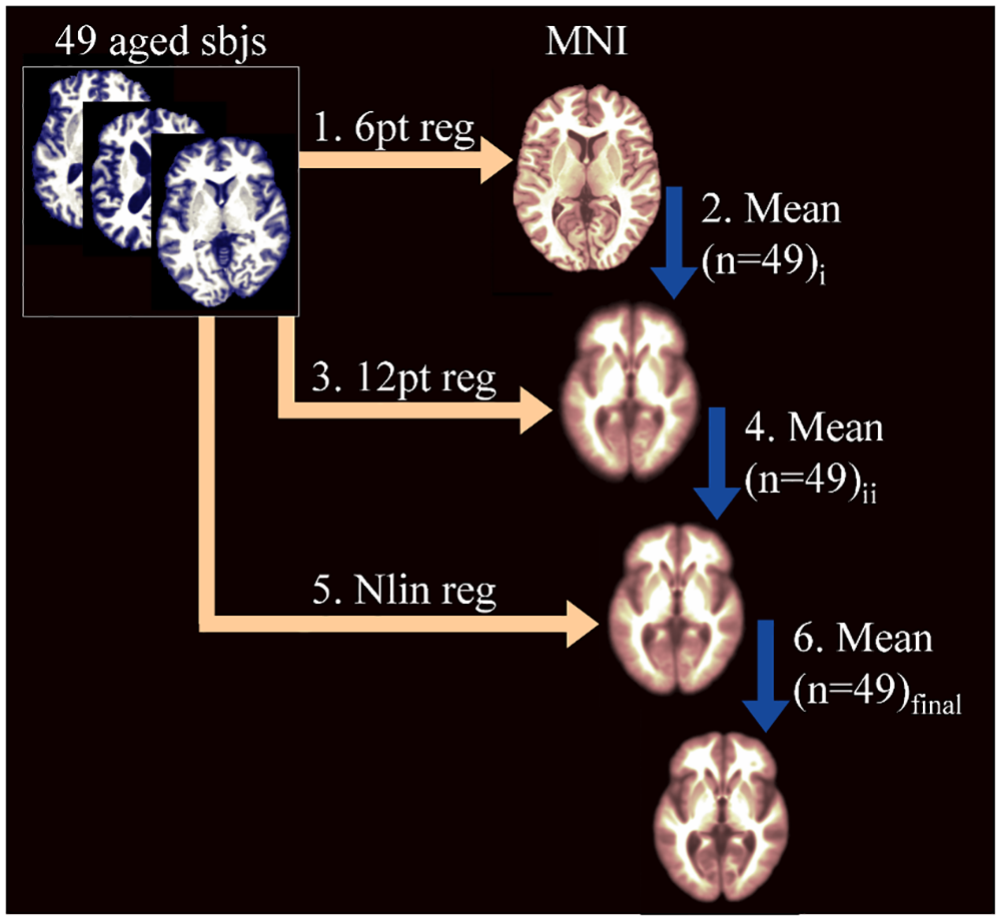
Procedure to create a brain MRI standard space representative of aged (>60 years) subjects. This is in the orientation of the commonly used Montreal Neurological Institute (MNI) template but reflects features of brain ageing, e.g. larger ventricles; sbjs = subjects; pt = point; reg = registration; Nlin = nonlinear.

Forty-nine subjects randomly selected from the normal group were used to create the registration target (standard space; Fig 1) by the following steps:

1. The brain extracted image of each randomly selected normal subject was orientated using a 6 point linear configuration (not stretched) to the MNI Colin27 brain extracted template (http://www.bic.mni.mcgill.ca/ServicesAtlases/HomePage).
2. The mean (n = 49) voxel-wise intensity of 6 point linear orientated normal brains was calculated to create a 6 point “aged” brain
3. Each of the 49 randomly selected normal subjects were then 12 point oriented and linearly deformed to the 6 point “aged” brain derived in step 2.
4. The mean (n = 49) voxel-wise intensity of 12 point linear orientated brains was calculated to create a 12 point “aged” brain
5. Each of the 49 randomly selected normal subjects were then nonlinearly deformed [38] to 12 point “aged” brain derived in step 4
6. The mean (n = 49) voxel-wise intensity of nonlinearly deformed brains was calculated to define the final “aged” brain standard space (Fig 1).

Fig 1 illustrates that our standard space (derived from 49 randomly selected normal older subjects) reflected the larger ventricles, sulcal spaces, and overall reduced brain tissue volume associated with aged brains (see Introduction for references describing brain structure in ageing). This standard space was used to derive the parametric (mean ±SD) and nonparametric (order-based) GM proportion atlas.

### 2.4 Spatial normalisation to standard space

To spatially normalise all subjects to our standard space, we devised *“nonlinear surface”* spatial normalisation (Nsurf). *Linear* spatial normalisation maintains within brain variance, e.g. ventricle size, between subjects but does not always adequately account for head size differences. *Nonlinear* spatial normalisation generally accounts for head size differences but removes within brain variance between subjects.

**Fig 2:**
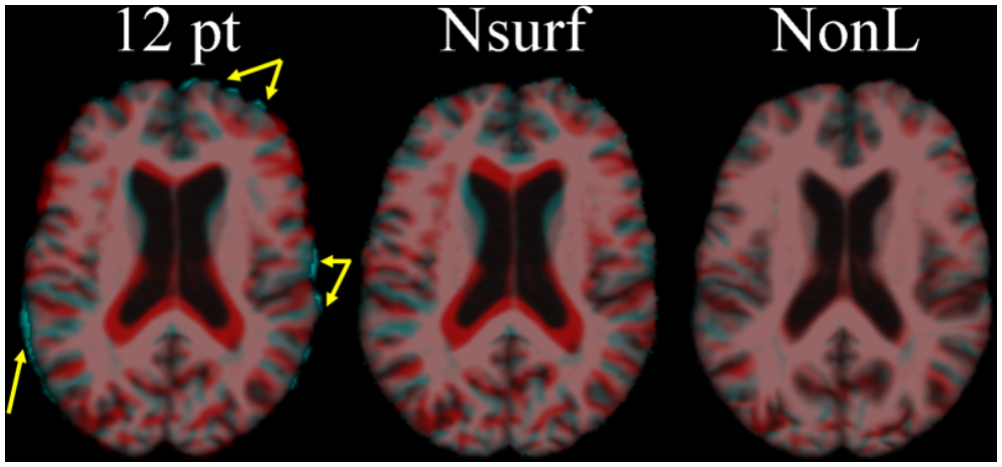
Nonlinear surface (Nsurf—middle panel) normalisation compared to conventional linear (12 pt—left panel) and nonlinear (NonL—right panel) normalisation. Two subjects (one cyan, the other red) were overlaid after each normalisation to the atlas standard space. The yellow arrows highlight differences in head size not accounted for by 12 pt normalisation. Most of the variance between subjects was removed in conventional NonL normalisation (note especially the lateral ventricles in the centre of the brain).

It is important to maintain within brain variance because, if for example we used conventional nonlinear registration, then all subject ventricles would be warped to the same size (Fig 2). The pathology we were interested in classifying, e.g., larger ventricles, would be removed prior to classification if we used conventional nonlinear registration. Further, unlike previous atlases, e.g., MNI (http://www.bic.mni.mcgill.ca/ServicesAtlases/HomePage), used for localisation and segmentation, the atlases that we describe are attempting to define variance in brain structure across individuals.

We therefore performed Nsurf by nonlinearly registering [38] each subject’s brain mask (binary image) to the standard space mask. Nonlinear transformations calculated with the brain masks (binary images) were then applied to original MRI and GM proportion images of each subject. This Nsurf spatial normalisation fully accounted for head size differences while also maintaining the within brain variance of interest (Fig 2). We assessed the effect of Nsurf quantitatively by calculating lateral ventricle volume [38] and cortical thickness [40] before and after normalisation.

#### 2.4.1 Effect of Nsurf spatial normalization

**Fig 3:**
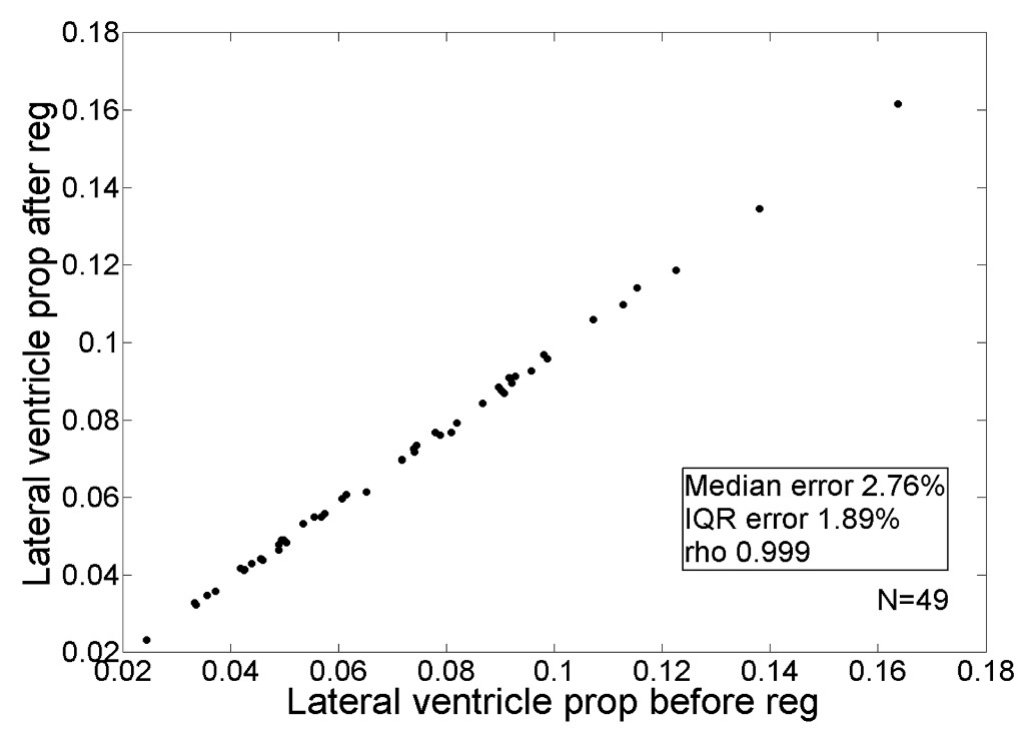
The effect of nonlinear surface normalisation (reg) on lateral ventricle volume expressed as a proportion (prop) of intracranial volume.

The result of Nsurf spatial normalisation, compared to conventional linear (12 pt) and nonlinear (NonL) normalisation, is illustrated in Fig 2. We qualitatively observed that Nsurf spatial normalisation accounted more adequately for head size differences than conventional 12 pt linear registration (Fig 2). Although it also appeared that the within-brain structure was maintained (compared to conventional nonlinear registration), we tested this quantitatively by computing ICV normalised lateral ventricle volumes before and after normalisation. The nominal error (median = 2.76%, IQR = 1.89%) in ICV normalised lateral ventricle volume following normalisation indicated that Nsurf did adequately maintain within-brain variance (Fig 3).

**Fig 4:**
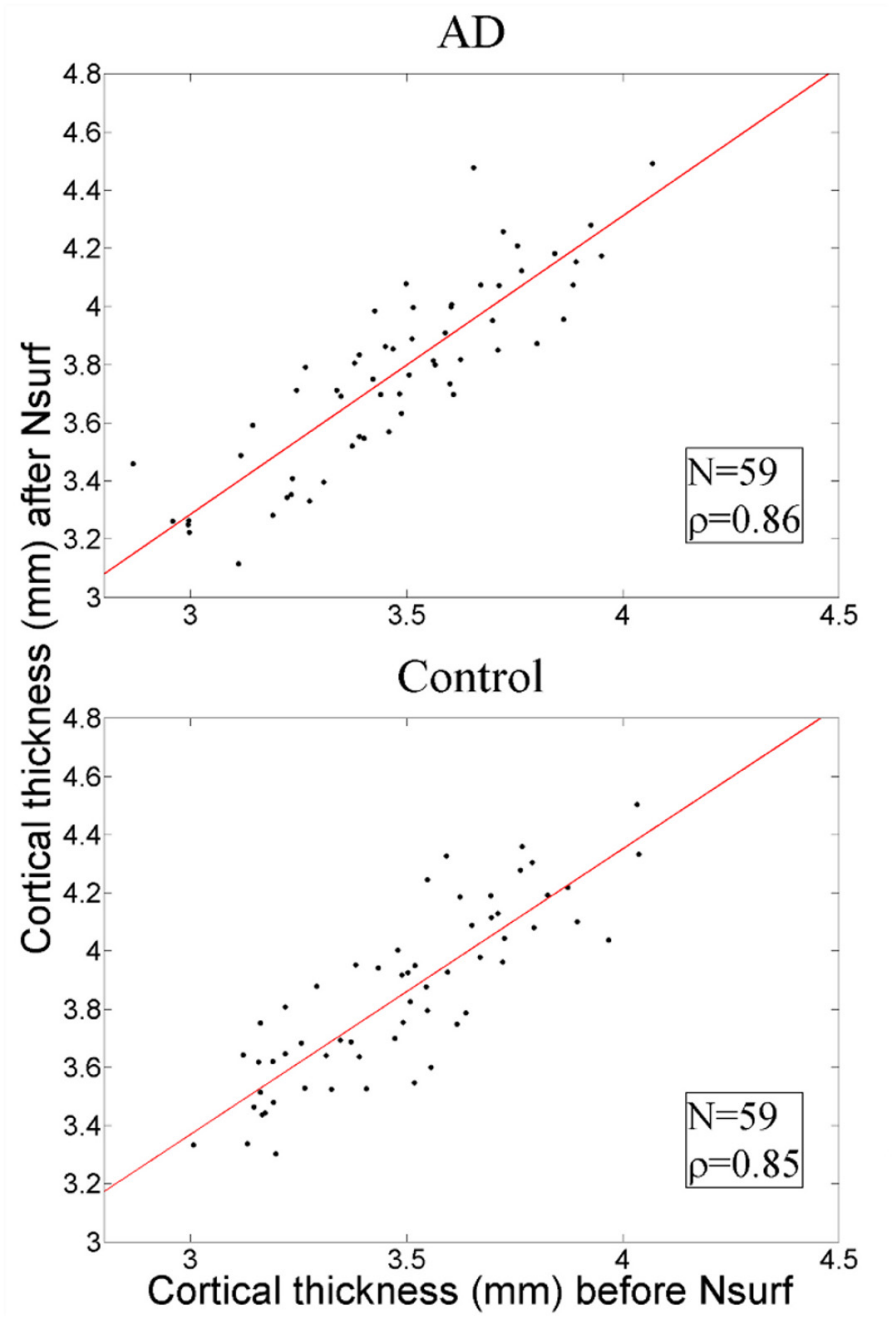
Cortical thickness before and after nonlinear surface (Nsurf) registration in AD subjects (top panel) and the matched control subjects (bottom panel). Cortical thickness was increased to the same level in both groups (as expected given the slightly larger standard space) and therefore Nsurf did not create a bias that would influence subsequent analyses.

Thus, inner parts of the brain (e.g., lateral ventricles) were not unduly altered by Nsurf. However, the Nsurf algorithm, by focusing on the surface of the brain, may have had more effect on cortical thickness. Fig 4 shows that, while Nsurf increased cortical thickness (as expected given that the standard space was slightly larger than most subjects), it did so to the same extent in both groups (ρ_AD_ = 0.86, ρ_Control_ = 0.85). In other words, Nsurf did not introduce bias that would influence subsequent analyses.

### 2.5 Assessing voxel-wise GM distributions

Distributions of data are commonly assessed with the Kolmogorov—Smirnov test. This test does not describe the shape of data; it determines whether or not the population from which the data were derived had a Gaussian (or other defined) distribution. We therefore calculated kurtosis and skewness to not only determine whether or not voxel-wise GM proportions were Gaussian distributed, but to determine the shape of their distribution.

#### 2.5.1 Kurtosis

Kurtosis measures the central and outer appearance of a data distribution [41]. It is calculated by eq 1,

$$\frac{\sum_{i\;=\;1}^{n}{\left(x_{i}-\mu \right)}^{4}}{\sigma^{4}}$$ (1)

where *x*_i_ are all the values of variable *x*, *μ* is the mean of variable *x*, and σ is the standard deviation of variable *x*. The Gaussian distribution has kurtosis of 3 [41].

#### 2.5.2 Skewness

Skewness measures the symmetry of a data distribution. It is calculated by eq 2,

$$\frac{\sum_{i\;=\;1}^{n}{\left(x_{i}-\mu \right)}^{3}}{\sigma^{3}}$$ (2)

where *x*_i-n_ are all the values of variable *x*, *μ* is the mean of variable *x*, and σ is the standard deviation of variable *x*. The Gaussian distribution has skewness of 0. After calculating voxel-wise kurtosis and skewness of GM, we created mean ±SD (parametric) and order-based (nonparametric) atlases of GM.

### 2.6 Parametric and nonparametric atlases of normal ageing GM proportion

GM proportion images from 98 randomly selected normal subjects (49 from ADNI and 49 from OASIS) were used to create parametric (mean ±SD) and nonparametric (order-based) atlases.

#### 2.6.1 Parametric (mean ±SD) GM atlas

In parametric (mean ±SD) based methods, percentile ranks and limits of distributions are approximated by eq 3,

$$\begin{array}{l} p\\ 2.5^{th}\;\mu –(2\times \sigma )\\ 25^{th}\;\mu –\;(0.7\times \sigma )\\ 50^{th}\;\mu \\ 75^{th}\;\mu +\;(0.7\times \sigma )\\ 97.5^{th}\;\mu +\;(2\times \sigma ) \end{array}$$ (3)

where *p* is the percentile rank, μ is the mean, and σ is the SD.

We used ±2 SD because it is a more generalisable estimate than the strict ±1.96 SD from the Normal distribution table [42], which may artificially inflate errors.

If data follow the Gaussian distribution, parametrically estimated values of percentile ranks should be approximately equal to nonparametric (order-based) values, e.g., the mean and median (50^th^ percentile) are equal in Gaussian distributions [42–44].

#### 2.6.2 Nonparametric (order-based) GM atlas

Nonparametric (order-based) methods calculate percentile rank values by eq 4,

$$\begin{array}{l} np\;=\;j+g\\ y\;=\;\frac{1}{2}(x_{j}+x_{j}{}_{+\;1})\;\;\;\text{if}\;g=0\\ y\;=x_{j}{}_{+\;1}\;\;\;\text{if}\;g>0 \end{array}$$ (4)

where *n* is the number of subjects, for the *t*th percentile *p* = *t*/100, *j* is the integer part of *np*, *g* is the fractional part of np, *y* is the *t*th percentile, and *x*_1_, *x*_*2*_,…, *x*_*n*_ are the ordered values of each brain volume.

### 2.7 Atlas based assessment

**Fig 5:**
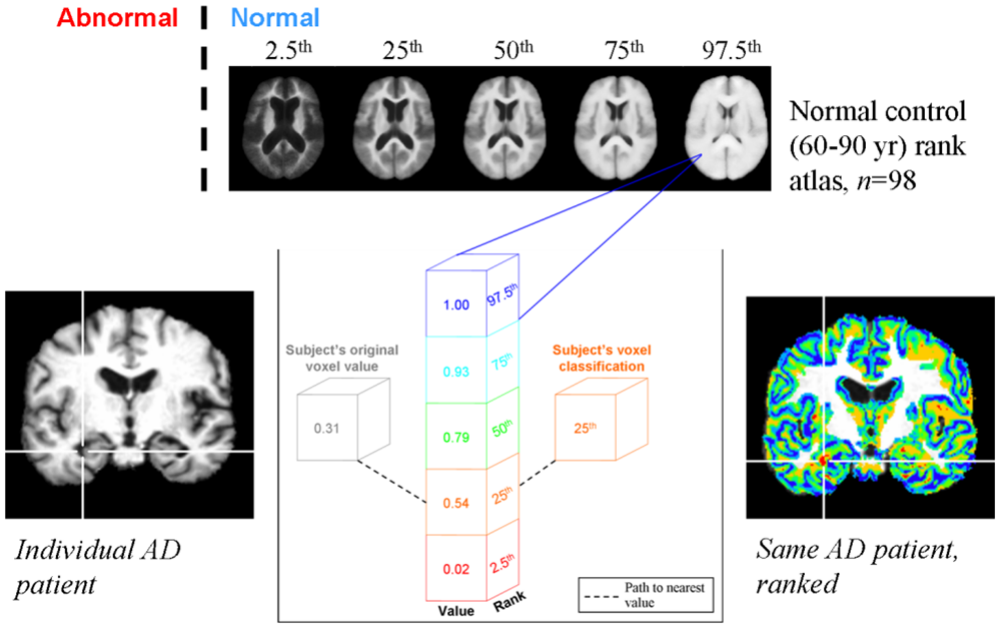
Voxel—based assessment of brain structure with a normal reference atlas. The subject's original voxel value is compared to each of the atlas percentile rank values and is then assigned the rank to which its value is nearest.

We used each atlas separately for quantitative, voxel-by-voxel assessment of the proportion of GM in individual AD patients. In each voxel in standard space, the proportion of GM in the AD subject was compared to the normal control atlas, and then assigned to the rank that was closest to their proportion of GM (Fig 5). Proportions of GM in individual subjects that were lower than the 2.5^th^ percentile in the normal control atlas were classified as “abnormal”.

According to the method illustrated in Fig 5, each subject was compared to the parametric (mean ±SD) atlas; and, separately, to the nonparametric (order-based) atlas.

We then identified voxels were there were differences in abnormal classifications between parametric and nonparametric atlases. Because of the apparently lobular weighted pattern of atrophy in ageing and AD (e.g., [17]), we report voxel-wise differences between methods by the major lobes (frontal, temporal, parietal, occipital).

## Results

We assessed voxel-wise distributions of GM proportion in the normal ageing atlas subjects, before constructing mean ±SD (parametric) and order-based (nonparametric) GM atlases.

### 3.1 Voxel-wise GM distributions in the normal atlas subjects

**Fig 6:**
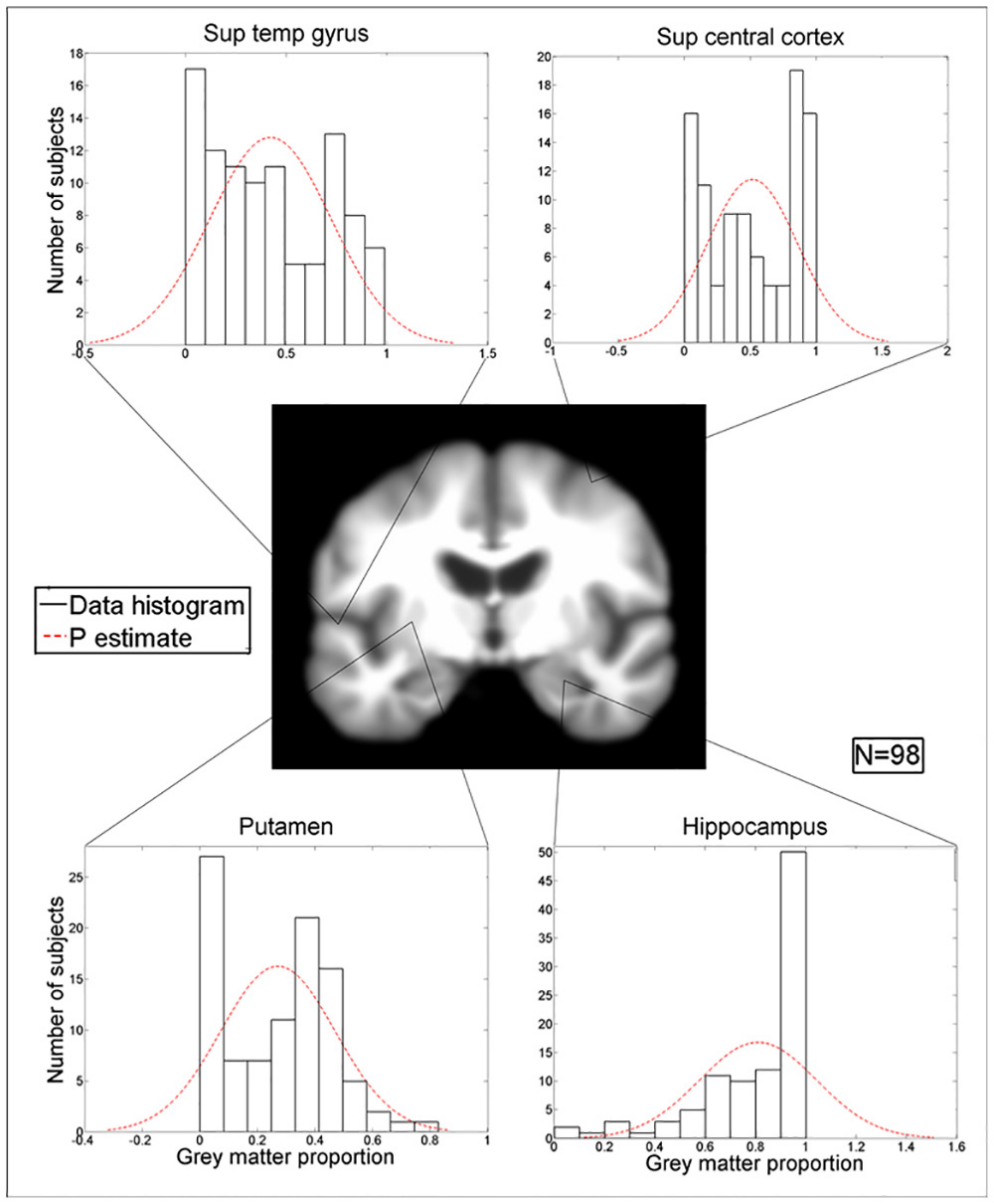
Distribution of the proportions of grey matter in 98 normal subjects (aged 60–90 years) at randomly selected voxels in the superior temporal gyrus (top left), superior central cortex (top right), putamen (bottom left), and hippocampus (bottom right). These distributions are markedly different to the assumed Gaussian (P) distribution (red dashed line).

Randomly selected examples of GM proportion distributions in the cortex and subcortical regions are shown in Fig 6.

**Fig 7:**
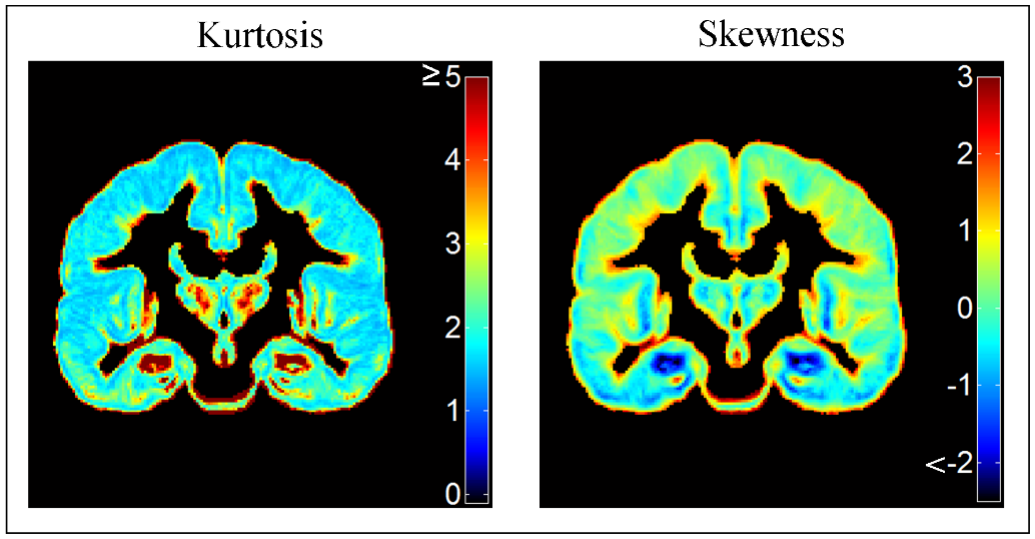
Voxel-wise kurtosis and skewness in the grey matter proportion atlas subjects (n = 98). The Gaussian distribution has kurtosis of 3 (yellow on the left panel) and skewness of 0 (light green on the right panel).

Fig 6 shows qualitatively that in these randomly selected example voxels, the distributions of the proportion of GM are markedly non-Gaussian. To assess GM distributions quantitatively throughout the cortex, we calculated kurtosis and skewness in each voxel with 98 subjects (Fig 7).

Fig 7 shows that kurtosis and negative skewness was greatest in the hippocampal region (dark red on left panel, dark blue on right panel). Median kurtosis across the entire cortex was 1.95 (interquartile range 0.95) and median skewness was 0.11 (IQR 0.92). Truly Gaussian data have kurtosis 3 (yellow on the left panel in Fig 7) and skewness 0 (light green on the right panel in Fig 7).

### 3.2 Parametric and nonparametric atlases of GM proportion

**Fig 8:**
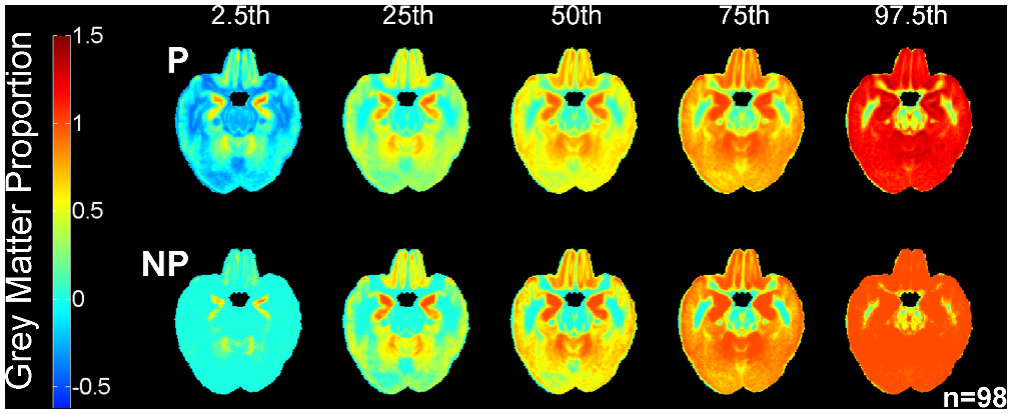
Atlases of the distribution of the proportions of GM in normal older subjects. These were calculated with parametric (mean ±SD; P—upper panel) and nonparametric (order-based; NP—lower panel) methods in 98 aged normal subjects (60–90 years)

**Fig 9:**
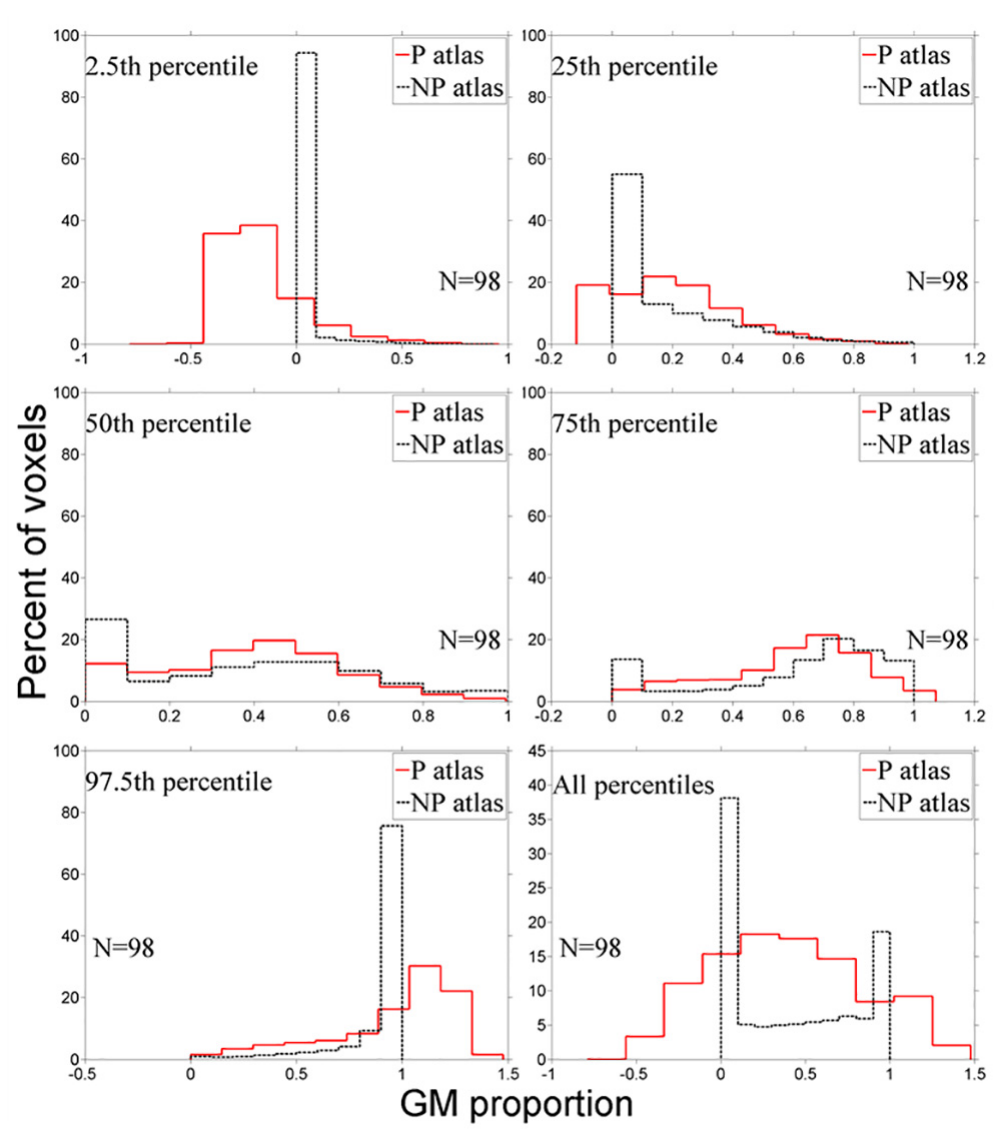
Histograms of the parametric (mean ±SD; P) and nonparametric (order-based; NP) grey matter (GM) atlas percentile ranks.

The parametric (mean ±SD) and nonparametric (order-based) atlases of the proportions of GM in normal older subjects (n = 98; 60–90 years) are shown in Fig 8. The parametric atlas often defined the lower limit of the normal (for age) proportion of GM as *less than* zero and the upper limit of normal (for age) as *greater than* one (top panel Fig 8). As GM proportion cannot be less than zero or more than 100%, this does not make sense mathematically or physiologically and is therefore an undesirable consequence of using the mean ±SD (parametric) method. The “true” limits are from zero to one, shown in the nonparametric (order-based) atlas (bottom panel Fig 8). Further differences between the atlases are illustrated by the histograms of voxel values in Fig 9.

Visually apparent differences between the parametric and nonparametric atlases are quantified in Table 1. Although they are often thought to be approximately equal [42,43], the mean (parametric 50^th^ percentile) and median (nonparametric 50^th^ percentile) histograms differed in skewness by more than 200%. The two methods agreed most closely at the 75^th^ percentile, but even here there were differences in the proportion of GM by an average of 52%.

**Table 1:** Kurtosis and skewness in parametric and nonparametric atlas histograms of the proportion of grey matter in whole brain voxels.

Percentile	Kurtosis			Skewness		
	*Parametric (mean*±*SD)*	*Nonparametric (order-based)*	*%Err*	*Parametric (mean*±*SD)*	*Nonparametric (Order-based)*	*%Err*
2.5^th^	5.99	33.76	–464	1.55	5.27	–240
25^th^	3.29	4.63	–41	0.75	1.45	–93
50^th^	2.45	2.04	17	0.06	0.20	–233
75^th^	2.53	2.42	4	–0.52	–0.79	–52
97.5^th^	3.43	10.31	–201	–1.13	–2.69	–138

Note: %Err = percent error between Gaussian and rank-based atlases

The greatest differences between atlases were at the lower limit (2.5^th^ percentile) of the normal proportion of GM for age. This led to differing classifications between atlas methods in many voxels (>25%) when used to assess subjects diagnosed with AD.

### 3.3 Parametric versus nonparametric atlas classifications in AD patients

For approximately half of the subjects in both AD samples, 25–45% of voxels that were classified as *abnormal* by the nonparametric atlas were classified as *normal* by the parametric atlas (“*normal P*, *abnormal NP*”; Table 2). Moreover, approximately 40–50% of voxels that were classified as *abnormal* by the parametric atlas were classified as normal by the nonparametric atlas (“*abnormal P*, *normal NP*”).

**Table 2:** Median percentage of differing classifications in AD subject voxels.

Classification	ADNI (*median*, *IQR*)	OASIS (*median*, *IQR*)
Normal P, abnormal NP	32.2, 8.2%	34.1, 9.3%
Abnormal P, normal NP	48.4, 6.2%	45.5, 6.6%

Note: ADNI = Alzheimer’s Disease NeuroImaging Initiative; OASIS = Open Access Series of Imaging Studies; IQR = interquartile range; P = parametric; NP = nonparametric.

**Fig 10:**
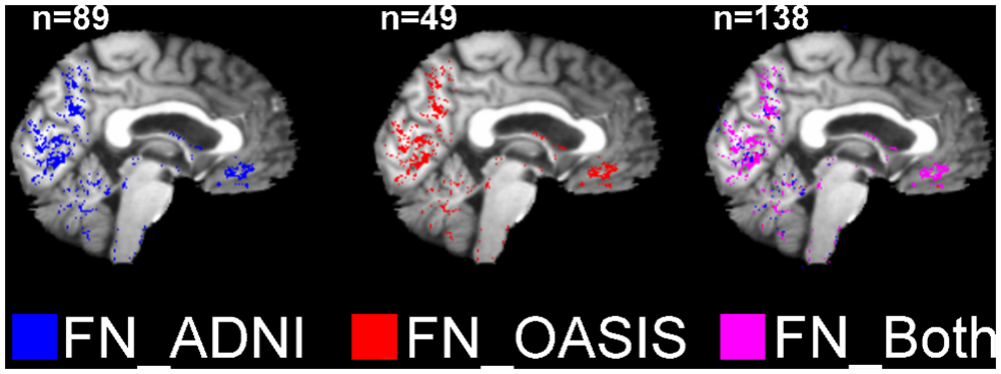
Locations of “*normal P*, *abnormal NP*” (FN) voxels in Alzheimer’s disease (AD) subjects (aged 60–90 years) from ANDI (n = 89) and OASIS (n = 49). These were determined via voxel assessment with the parametric (mean±SD) and nonparametric (order-based) grey matter atlases; P = parametric; NP = nonparametric; ADNI = Alzheimer’s Disease Neuroimaging Initiative; OASIS = Open Access Series of Imaging Studies.

**Fig 11:**
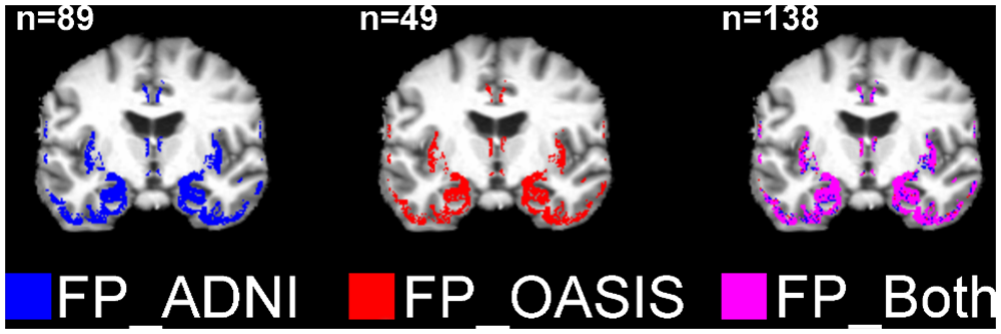
Locations of “*abnormal P*, *normal NP*” (FP) voxels in Alzheimer’s disease (AD) subjects (aged 60–90 years) from ANDI (n = 89) and OASIS (n = 49). These were determined via voxel classification with parametric (mean±SD) and nonparametric (order-based) grey matter atlases; P = parametric; NP = nonparametric; ADNI = Alzheimer’s Disease Neuroimaging Initiative; OASIS = Open Access Series of Imaging Studies.

Proportions of “*normal P*, *abnormal NP*” classifications were approximately equal between the frontal and temporal lobes (Table 3). There were concentrated regions of “*normal P*, *abnormal NP*” classifications in the mid saggital caudal and anterior regions, in both samples (Fig 10). Approximately two thirds of “*abnormal P*, *normal NP*” classifications occurred in the temporal lobe (Table 3), particularly in the hippocampal region (Fig 11).

**Table 3:** Proportions of differing classifications by lobe.

		Frontal	Temporal	Parietal	Occipital
Normal P, abnormal NP	ADNI	0.36	0.38	0.09	0.17
	OASIS	0.39	0.41	0.08	0.12
Abnormal P, normal NP	ADNI	0.28	0.57	0.06	0.08
	OASIS	0.27	0.61	0.06	0.06

Note: P = parametric; NP = nonparametric; ADNI = Alzheimer’s Disease Neuroimaging Initiative; OASIS = Open Access Series of Imaging Studies. Rounding errors mean that not all rows in this table sum to exactly 1.

Tables 2 and 3 and Figs 10–11 show that the incidences of differences between parametric and nonparametric classifications were well replicated across both independent samples (ADNI and OASIS).

### 3.4 Simulated non-Gaussian data

GM distributions were often similar to the Poisson distribution (Fig 6). We therefore used simulated Poisson data to provide further evidence of differences between parametric and nonparametric methods in non-Gaussian data. Table 4 shows that parametric and nonparametric methods are similar in central percentile ranks, e.g., mean versus median, but diverse towards extreme percentile ranks, e.g., 2.5^th^ order versus mean-2*SD. This suggests that parametric estimates are generally robust for estimating central tendencies, e.g., means, even in non-Gaussian data.

**Table 4:** Parametric and nonparametric percentile rank values in simulated non-Gaussian data.

Percentile rank	2.5^th^		25^th^		50^th^		75^th^		97.5^th^	
	P	NP	P	NP	P	NP	P	NP	P	NP
N = 98	-2.16	0.00	0.65	1.00	2.16	2.00	3.68	3.00	6.49	6.00
N = 10000	-1.98	0.00	0.60	1.00	1.98	2.00	3.37	3.00	5.95	5.00

Note: N = number of points in randomly generated data; P = parametric; NP = nonparametric. These data are from Poisson distributions that were randomly generated using the “poissrnd” function in MATLAB.

Increasing the simulation sample size from 98 to 10000 brings the parametric and nonparametric estimates of extreme percentile ranks slightly closer but only by approximately 10% (Table 4). The minimum value in either randomly generated sample was zero but the parametric method estimated the 2.5^th^ percentiles to be negative. This is consistent with our subject data that suggest nonparametric methods may be more robust for defining the limits of brain structure if large amounts of data are available.

## Discussion

To our knowledge, we have presented the first nonparametric brain MRI atlas of the proportions of GM in normal older subjects (≥60 years). Up until now, many MRI atlases of the normal brain were designed for image registration and other pre-processing steps. These were predominantly for use in studies of young subjects, and were derived using parametric (e.g., mean ±SD) methods [21,24]. Parametric estimates are generally robust for defining central tendencies, e.g., means, of brain structure. But brain MRI voxel data (e.g., the proportion of GM) must approximate a Gaussian distribution to reliably extrapolate parametric values (e.g., mean ±SD) and define the limits of normal brain structure [42,43].

We have shown that the voxel-wise proportions of GM in normal older subjects aged ≥60 years often did not follow a Gaussian distribution. This led to nonsensical estimates of the lower limits of GM proportion by the parametric atlas, e.g., the parametric atlas estimated the lower limit of GM proportion to be negative in many voxels. Approximately 70% of the cortex and subcortical GM in normal older subjects (≥60 years) had either high kurtosis and/or negative skewness. The largest deviations from the Gaussian distribution were in temporal and frontal regions, which are both important regions that are affected early in dementias [16]. This resulted in many differing voxel classifications between parametric and nonparametric atlases when used to assess subjects diagnosed with AD.

For approximately half of the subjects in both AD samples, 25–45% of voxels that were classified as abnormal by the nonparametric atlas were classified as normal by the parametric atlas. The pattern of differing classifications in AD subjects according to statistical method was repeated in two independent samples (ADNI and OASIS).

Voxels classed as abnormal in the parametric atlas but normal according to the nonparametric atlas were largely in the temporal lobe (~60%), and specifically focused in the hippocampus. This suggests that this region may have a wide and irregular variance and be particularly unsuited to parametric Gaussian analyses in older subjects. Moreover, many (~40%) of voxels classed as normal in the parametric atlas but abnormal according to the nonparametric atlas were found in frontal and occipital regions. Although less often reported in research, frontal-occipital pathology is often noted in patients diagnosed clinically with AD and other dementias [45].

Although nonparametric, order-based methods are said to provide “true” percentile rank values [42], these values are only true in the samples from which they were derived. In other words, the nonparametric percentile rank values calculated here do not necessarily reflect population values. Although we used “parametric” and “Gaussian” somewhat interchangeably, parametric models do not always use Gaussian distributions and Gaussian models do not need to be parametric. For example, Gaussian mixture models have been proposed to estimate brain MRI distributions in younger subjects [28], but do not seem suitable for the highly skewed distributions we found in older subjects (e.g., Fig 6). Our work emphasises that large amounts of brain MRI data from normal older subjects are required to define normal ageing population distributions. Hence we are collecting such data now and encourage others to join our initiative (http://www.sinapse.ac.uk/research-resources/brains-project). With these large volumes of data we may be able to propose distributions that more accurately reflect underlying brain image data distributions, e.g., mixture or Poisson distributions.

There appears to be a reasonably well defined lobar pattern of atrophy in ageing and neurodegenerative disease [14,15,17], therefore we reported differences in voxel-wise classifications by automated segmentation of the major lobes. We performed manual checking for gross errors, but minor errors were not edited due to the impracticality of manually editing hundreds of segmentations consisting of small isotropic voxels. Moreover, we could not tabulate voxel-wise differences in classifications for subregional volumes, e.g., the hippocampus. Further work is required to improve the accuracy and efficiency of sub-lobar segmentations in older subjects [46].

We did not implement other brain MRI processing methods that are commonly used in younger subjects. In particular, we did not apply voxel smoothing, nor use conventional nonlinear registration (we used our own “Nsurf” normalisation) for the following reasons. We did not use conventional nonlinear registration because we wished to maintain the naturally wide anatomical variance in the brains of normal older people. Voxel smoothing may mask subtle differences between normal and diseased brains because it essentially “averages” the intensities of adjoining voxels [2,3,9,11]. Moreover, smoothing is a subjective process with no quantitatively derived optimal parameters as yet [47]. This means that we currently do not know how the distributions of voxels in the ageing brain will behave after conventional nonlinear registration and smoothing. Our finding that the proportions of GM are least Gaussian in the hippocampus is in conflict with a previous study that found the hippocampus voxel data follow an approximately Gaussian distribution [26]. However, the 85 subjects in that study were much younger (mean age ~31 years, SD ~8.0; range: 17–60), and underwent voxel smoothing, which makes data more Gaussian [48,49].

We have presented a nonparametric method that does not require data to be Gaussian, and therefore an alternative method when the use of smoothing cannot be justified. For example, when looking for subtle differences in brain structure in individual subjects at the extremes of life and/ or on the cusp of overt neurodegenerative disease [2,3,8,11,15].

We defined the normal limits of GM proportion in older people from 98 randomly selected normal control subjects in ADNI (n = 49) and OASIS (n = 49) that were aged between 60 and 90 years. These data were cross sectional and may therefore contain sampling biases. There are large differences in brain volume between these ages [1–7]. This number of subjects is not sufficient to define population values but is consistent with previous studies of brain structure in ageing [34]. Moreover, percentile rank values became stable after approximately 70 subjects were added to the nonparametric atlas (shown via oscillation analysis; S1 Fig in Supporting Information). Future work will aim to create separate atlases for each year of life. Further, we will incorporate other clinical and cognitive data into these atlases, e.g. atlases for specific blood pressures and/ or cognitive ability. We have created an atlas and assessment system that is designed, via percentile rank classification, to highlight potentially problematic areas of brain structure in individual patients. This system is not designed to classify individual patients into groups, e.g., AD versus control, which has been done previously [51].

We illustrated our novel atlas-based assessment method using publicly available images of the proportions of GM from normal older subjects and subjects diagnosed with AD. We did not test our atlases in the remaining normal subjects with T1-weighted images from ADNI (total N = 138) and OASIS (total N = 98) because this is the focus of another report currently in preparation. However, the software we developed may be applied to any image sequence from any group. For example, we intend to create a T1–weighted percentile rank atlas from full term infants to assess brain structure in preterm infants [50]. Our software and data from over 1000 cognitively tested normal subjects (0–90 years) will be made available via the BRAINS bank (http://www.sinapse.ac.uk/research-resources/brains-project). Our software was coded in MATLAB so may easily be implemented into commonly used brain MRI processing pipelines, e.g. Statistical Parametric Mapping (SPM).

Assessments of brain MRI from individual subjects are becoming increasingly important as AD and other neurodegenerative diseases become a major public health burden [12,13]. Comparison of individual scans with atlases of normal older subjects may assist in future to diagnose faster-than-usual brain tissue loss in prodromal dementia, or to diagnose types of established dementia by differentiating patterns of abnormal brain tissue. We have demonstrated that much of the cortex and subcortical GM voxels are not distributed approximately Gaussian in normal ageing. We therefore conclude that nonparametric atlases may be useful when assessing possible neurodegenerative disease in older age.

## Supporting Information

S1 Fig Determining the number of subjects required to recreate a complete nonparametric atlas representative of the total sample n = 98 nonparametric atlas. Each coloured line represents the change in each percentile rank value (2.5^th^—97.5^th^) given the addition of more subjects. Seventy subjects (~71%) were required to create a nonparametric atlas that was 95% similar to the total *n* = 98 nonparametric atlas (shown by the dashed vertical line) and 90 subjects (~92%) were required to create a nonparametric atlas that was 99% similar to the total *n* = 98 nonparametric atlas (shown by the solid vertical line).
(TIF)

S1 Text Oscillations of percentile rank values in the nonparametric atlas, given the number of subjects, are shown in S1 Fig. This shows the percent similarity of nonparametric atlas histograms (with *n* = 10, 20, …, 98 subjects) to the total *n* = 98 nonparametric atlas histogram. Oscillations in percentile rank values were limited after 70 subjects had been added to the nonparametric atlas (S1 Fig).
(DOCX)
