# Supplementary figures and images for: Use of Brain MRI Atlases to Determine Boundaries of Age-Related Pathology: The Importance of Statistical Method
Source: PLoS One. 2015 May 29;10(5):e0127939. doi: 10.1371/journal.pone.0127939 (PMC4449178; doi:10.1371/journal.pone.0127939)

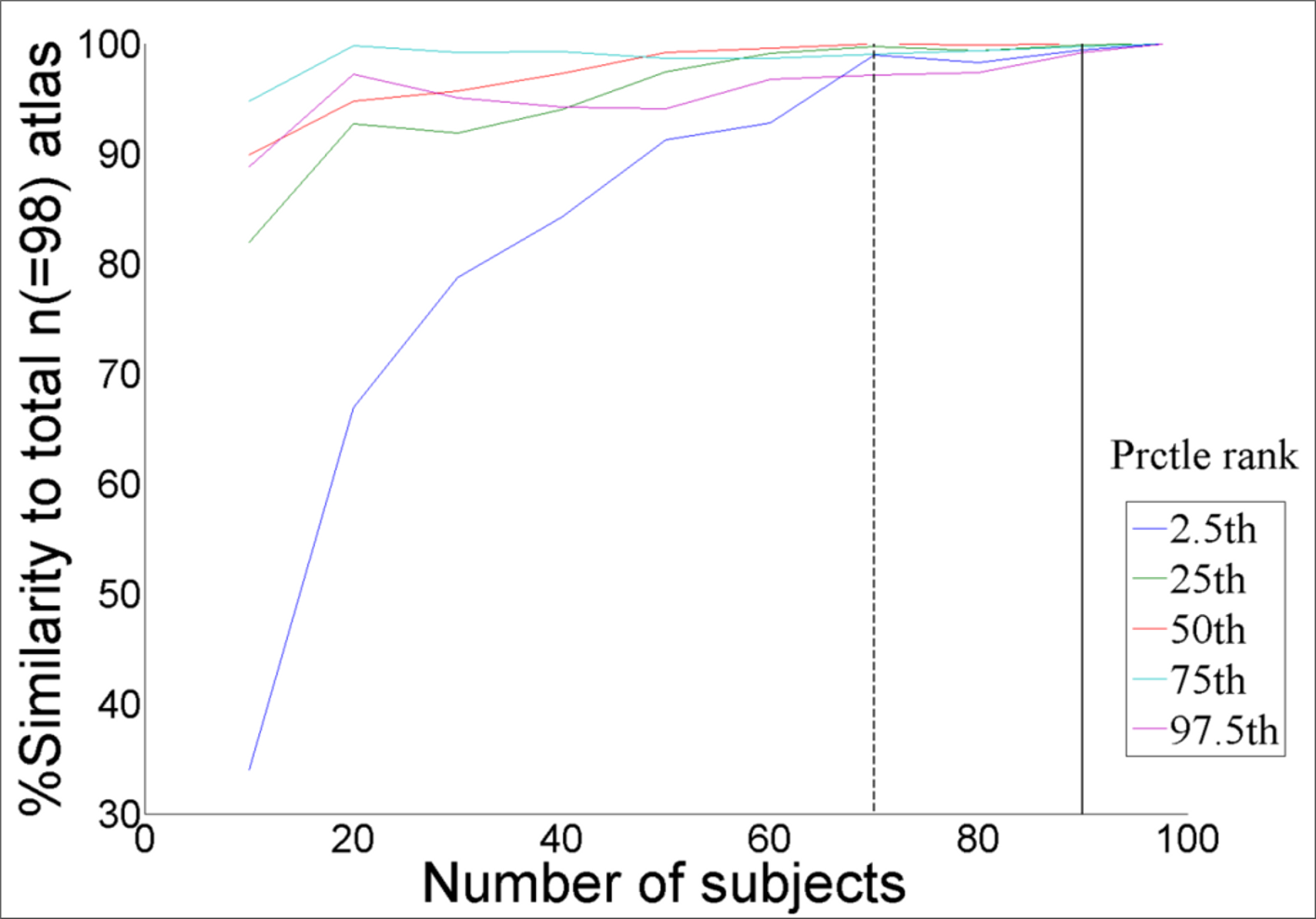

Supplement: S1 Fig — Each coloured line represents the change in each percentile rank value (2.5th—97.5th) given the addition of more subjects. Seventy subjects (~71%) were required to create a nonparametric atlas that was 95% similar to the total n = 98 nonparametric atlas (shown by the dashed vertical line) and 90 subjects (~92%) were required to create a nonparametric atlas that was 99% similar to the total n = 98 nonparametric atlas (shown by the solid vertical line). (TIF) [file pone.0127939.s001.tif]
