# Supplementary material for: Use of Brain MRI Atlases to Determine Boundaries of Age-Related Pathology: The Importance of Statistical Method
Source: PLoS One. 2015 May 29;10(5):e0127939. doi: 10.1371/journal.pone.0127939 (PMC4449178; doi:10.1371/journal.pone.0127939)
Supplement: S1 Text — This shows the percent similarity of nonparametric atlas histograms (with n = 10, 20, …, 98 subjects) to the total n = 98 nonparametric atlas histogram. Oscillations in percentile rank values were limited after 70 subjects had been added to the nonparametric atlas (S1 Fig). (DOCX) [file pone.0127939.s002.docx]

**Supporting Information**

Oscillations of percentile rank values in the nonparametric atlas, given the number of subjects, are shown in Figure S1. This shows the percent similarity of nonparametric atlas histograms (with *n*=10, 20, …, 98 subjects) to the total *n*=98 nonparametric atlas histogram. Oscillations in percentile rank values were limited after 70 subjects had been added to the nonparametric atlas (Figure S1).
